# Ear, nose and throat manifestations of autoimmune and autoinflammatory diseases: a rheumatology perspective

**DOI:** 10.1016/j.bjorl.2021.05.015

**Published:** 2021-10-22

**Authors:** Samuel de Oliveira Andrade, Simone Appenzeller

**Affiliations:** a Universidade de Campinas, Faculdade de Ciências Médicas, Pós-Graduação do Programa de Fisiopatologia, Campinas, SP, Brazil; b Universidade de Campinas, Faculdade de Ciências Médicas, Departamento de Ortopedia, Reumatologia e Traumatologia, Unidade de Reumatologia, Campinas, SP, Brazil

Ear, nose and throat (ENT) manifestations are an important hallmark of inflammatory rheumatic diseases, varying from mild to life-threatening manifestations. They can be the first symptoms or occur during the course of the disease. The correct identification of the underlying physiopathology (inflammation, thrombosis or infection) and proper treatment of ENT symptoms are therefore of importance in diagnosis and followup of patients with inflammatory rheumatic disease. Many classification criteria include ENT manifestations, such as granulomatosis with polyangiitis (GPA), Behçet disease (BD), relapsing polychondritis (RP), eosinophilic granulomatosis with polyangiitis (EGPA) and Cogan syndrome (GS). Other autoimmune/autoinflammatory diseases are characterized by widespread inflammation and may present with ENT symptoms, such as sarcoidosis, rheumatoid arthritis (RA), Sjogren syndrome (SS), systemic lupus erythematosus (SLE) and systemic sclerosis (SSc).^1^, ^2^, ^3^, ^4^, ^5^

In this editorial, we will review on the main ENT manifestations that have been described in inflammatory rheumatic diseases.

## Ear involvement

### Outer ear

Auricular chondritis is present in 20% at the onset of RP and 90% during the course of the disease. The entire ear is swollen, red and painful at contact. The continuous inflammation can result in pinna cauliflower deformity or ossification of the connective tissue. RP is characterized by a rare multisystem disease accepted as a complex autoimmune disorder affecting proteoglycan-rich structures and cartilaginous tissues, especially the auricular pinna, cartilage of the nose, tracheobronchial tree and various organ’s connective components. Auricular chondritis can also be observed in GPA. It is a rare autoimmune disorder characterized by granulomatous inflammation and small-vessel vasculitis associated with antineutrophil cytoplasmic antibodies (ANCA). GPA has a broad clinical spectrum that ranges from predominantly granulomatous manifestations restricted to the respiratory tract to severe, life-threatening necrotizing vasculitis.^1^, ^2^, ^3^

### Middle ear

Otalgia, secretory otitis media and otorrhea can be observed in GPA and imunoglobulin G4 related disease (IgG4-RD). IgG4-RD is a chronic inflammatory disease that involves many tissues such as pancreas, lacrimal and salivary glands. Ear manifestations are more common in the middle ear, and the inner ear is rarely affected. Recurrent mastoiditis and facial numbness can also be observed.^4^

### Inner ear

Sensorineural hearing loss (SNHL) can be observed in a variety of systemic autoimmune/autoinflammatory diseases, having a prevalence of 21-69% for RA, 8-28% for SLE, 21-46% for SS and 20-77% for systemic sclerosis. Possible mechanisms include disease related (vasculitis, thrombosis or antibody-mediated) and medication related (ototoxic effect). The presence of vestibuloauditory symptoms, as sudden onset tinnitus and vertigo that can present with nausea, vomiting, ataxia and nystagmus in association especially with non-infectious interstitial keratitis, but also other symptoms (conjunctivitis, uveitis, episcleritis, scleritis, optic neuritis) should raise the suspicion of Cogan Syndrome (CS).^1^, ^2^

SNHL in association with increased inflammatory markers and the presence of recurrent fever in the absence of infection should alert otolaryngologists to the possibility of autoinflammatory diseases. Systemic autoinflammatory diseases (SIAD) are a group of disorders caused by a dysregulation of the innate immune system. One of most common monogenic SIAD associated with hearing loss is mutation of NOD- like receptor gene (NLRP3), a condition known as NLRP3-associated autoinflammatory disease (NLRP3-AID), formerly known as Cryopyrin-associated periodic syndrome (CAPS). Muckle Wells syndrome (MWS) is the intermediate form of CAPS and can develop to progressive SNHL, secondary to chronic inflammation of the internal ear. SNHL in MWS often rapidly progresses from mild high tone deficits to complete deafness. Early hearing loss primarily affects high frequencies of > 6 kHz reflecting the characteristic high sensitivity pattern of hair cells to injury as described in other systemic conditions. Cochlear enhancement on fluid attenuation inversion recovery MRI (FLAIR- MRI) is more frequent in patients with a higher prevalence of hearing loss, providing some insight into the mechanisms of SNHL in MWS.^5^

## Nose and sinus involvement

Nasal involvement in GPA is present in approximately 42% of patients. Other associated symptoms related to GPA activity are nasal inflammation, chronic sinusitis, and nasal crusting with or without bloody rhinorrhea.

Nasal chondritis can affect up to 15% of patients of RP. The inflammation involves the bridge of the nose, causing nasal pain, redness and swelling, being less marked than the ears. Nasal obstruction is rare. Both RP and GPA can progress to permanent damage, such as characteristic ‘saddle-nose’ deformity or septal perforation.^1^

EGPA is a rare necrotizing ANCA-associated vasculitis. EGPA is characterized by the presence of asthma, peak blood eosinophilia and small-size vessel vasculitis. There are many nasal manifestations associated with the disease, such as chronic sinusitis, nasal crusting, allergic rhinitis and bilateral diffuse polyposis. The presence of these manifestations associated with vasculitis in biopsy or ANCA positivity should rise the suspicion of EGPA.

## Oral involvement

Sicca syndrome is by far the most frequent oral manifestation in autoimmune diseases, being present in more than 95% of patients with SS. Xerostomia may lead to secondary problems like oral candidiasis, dental caries and periodontal disease. In addition to that, sicca symptoms can lead to hoarseness and non-productive cough. Recurrent parotitis is present in 30-50% of patients and is characterized by a firm, diffuse, non-tender swelling. It is important in differentiate it from recurrent juvenile parotitis, where ANA antibodies are generally absent.

BD is a systemic vasculitis, affecting vessels of variable sizes. It is characterized by a myriad of systemic manifestations, including mucocutaneous, arthritis, vascular, neurological and gastrointestinal. Recurrent oral ulceration is the most frequent presenting ENT symptom (95% of patients), typically being multiple and variable size (2-20 mm) and occur extensively on the buccal membrane, tongue, palate and in the oropharynx. The ulcers are classically painful, surrounded by erythema and the larger ones heal with scarring. Six or more painful, recurrent ulcers, of variable size with surrounding erythema occurring on the soft palate or oropharynx should heighten suspicion of BD.^1^, ^2^

SLE mucous membrane involvement is characterized by oral ulcers. Classical lesions are asymptomatic, occur at the hard palate and are characterized by whitish plaques with erythema in the center and keratotic striae in the periphery with areas of telangiectasia.^1^, ^2^

## Throat involvement

Laryngeal chondritis is an important symptom of RP and it manifests as pain above the thyroid gland and dysphonia, causing laryngomalacia or stenosis in more severe cases. Hypoglottic stenosis, due to granulomatous inflammation of GPA occurs in 2-20% and is a potential life-threatening complication and is associated with systemic involvement. Dyspnea, voice changes and cough are the most common symptoms, and audible stridor is present in most severe cases.^1^

RA is a chronic inflammatory disease characterized by symmetrical inflammatory joint disease that may evolve to joint damage and bone destruction. Laryngeal manifestations of RA involve cricoarytenoid joint (CJ) arthritis and rheumatoid nodules. The symptoms of CJ arthritis in the acute stage are often fullness in the throat or a feeling of tension, in addition to hoarseness, odynophagia or dysphagia, as well as pain worsening by speaking. Chronic disease can be manifest by a husky voice and stridor.^1^, ^2^

In summary, inflammatory rheumatic are rare diseases that present with a wide spectrum of ENT manifestations. A high index of suspicion is necessary for timely diagnosis. ENT specialists have a crucial role in referring patients with possible inflammatory diseases to rheumatologists.

## Fundings

National Council for Scientific and Technological Development (CNPQ): 306723/2019-0.

Coordination of Superior Level Staff Improvement (CAPES): 001.

## Conflicts of interest

The authors declare no conflicts of interest.
